# Transcriptome and key genes expression related to carbon fixation pathways in *Chlorella* PY-ZU1 cells and their growth under high concentrations of CO_2_

**DOI:** 10.1186/s13068-017-0868-z

**Published:** 2017-07-11

**Authors:** Yun Huang, Jun Cheng, Hongxiang Lu, Yong He, Junhu Zhou, Kefa Cen

**Affiliations:** 1 State Key Laboratory of Clean Energy Utilization, Zhejiang University, Hangzhou, 310027 China; 2 Key Laboratory of Low-grade Energy Utilization Technologies and Systems of Ministry of Education, Chongqing University, Chongqing, 400044 China

**Keywords:** CO_2_ fixation pathway, Genes transcript sequences, 15% CO_2_ concentration, Carbonic anhydrase, Rubisco

## Abstract

**Background:**

The biomass yield of *Chlorella* PY-ZU1 drastically increased when cultivated under high CO_2_ condition compared with that cultivated under air condition. However, less attention has been given to the microalgae photosynthetic mechanisms response to different CO_2_ concentrations. The genetic reasons for the higher growth rate, CO_2_ fixation rate, and photosynthetic efficiency of microalgal cells under higher CO_2_ concentration have not been clearly defined yet.

**Results:**

In this study, the Illumina sequencing and de novo transcriptome assembly of *Chlorella* PY-ZU1 cells cultivated under 15% CO_2_ were performed and compared with those of cells grown under air. It was found that carbonic anhydrase (CAs, enzyme for interconversion of bicarbonate to CO_2_) dramatically decreased to near 0 in 15% CO_2_-grown cells, which indicated that CO_2_ molecules directly permeated into cells under high CO_2_ stress without CO_2_-concentrating mechanism. Extrapolating from the growth conditions and quantitative Real-Time PCR of CCM-related genes, the *K*_m_ (CO_2_) (the minimum intracellular CO_2_ concentration that rubisco required) of *Chlorella* PY-ZU1 might be in the range of 80–192 μM. More adenosine triphosphates was saved for carbon fixation-related pathways. The transcript abundance of rubisco (the most important enzyme of CO_2_ fixation reaction) was 16.3 times higher in 15% CO_2_-grown cells than that under air. Besides, the transcript abundances of most key genes involved in carbon fixation pathways were also enhanced in 15% CO_2_-grown cells.

**Conclusions:**

Carbon fixation and nitrogen metabolism are the two most important metabolisms in the photosynthetic cells. These genes related to the two most metabolisms with significantly differential expressions were beneficial for microalgal growth (2.85 g L^−1^) under 15% CO_2_ concentration. Considering the micro and macro growth phenomena of *Chlorella* PY-ZU1 under different concentrations of CO_2_ (0.04–60%), CO_2_ transport pathways responses to different CO_2_ (0.04–60%) concentrations was reconstructed.

**Electronic supplementary material:**

The online version of this article (doi:10.1186/s13068-017-0868-z) contains supplementary material, which is available to authorized users.

## Background

Global warming necessitates the reduction of accumulated CO_2_ in the atmosphere. Utilizing biological conversions by microalgae is a promising approach to reduce CO_2_ emissions [1, 2]. However, the current atmospheric CO_2_ concentration of ~0.04% is not enough for microalgae photosynthesis [3]. Moreover, the photosynthetic mechanism ribulose-1,5-bisphosphate carboxylase/oxygenase (rubisco), the first and stromal enzyme that catalyzes the entry of CO_2_ into the Calvin–Benson cycle, is adapted to the considerably higher CO_2_ concentrations encountered by C_3_ plants [4]. The *K*_m_ for CO_2_ of microalgal rubisco, often exceed 25 μM [5]. However, the dissolved CO_2_ in freshwater is only ~15 μM when in equilibrium with air. However, rubisco has a poor apparent affinity with CO_2_ when the dissolved CO_2_ concentration is less than the *K*_m_ (CO_2_) of rubisco, led to decrease in photosynthetic efficiency [6, 7]. To overcome this challenge, a number of prokaryotic and eukaryotic microalgae have developed a CO_2_-concentrating mechanism (CCM) to maximize photosynthesis under limited CO_2_ conditions [8]. Given that CO_2_ is the only form of dissolved inorganic carbon that rubisco can fix, the most likely evolutionary goal of the CCM process is to increase dissolved CO_2_ concentrations at rubisco locations for fixation [9].

A number of studies have been conducted on the mechanisms underlying the acclimation of microalgal cells to limited CO_2_ concentrations [7, 8, 10]. Carbonic anhydrase (CAs) and CCM play an important role in the efficient utilization of dissolved inorganic carbon under CO_2_-limited conditions. However, CCM requires more energy flow in PSI and leaves less energy available for the Calvin–Benson cycle, thus reducing microalgal growth and CO_2_ fixation efficiency [11]. Therefore, several studies have focused on CO_2_ fixation by microalgae from high concentrations of CO_2_ gas, such as flue gas, to increase CO_2_ fixation efficiency [12]. In our previous study, the biomass yield (2.78 g L^−1^) of *Chlorella* PY-ZU1 cultivated under 15% CO_2_ increased by 1.19-fold compared with that of microalgae cultivated under air (1.30 g L^−1^) [13]. However, less attention has been given to the photosynthetic mechanisms of microalgal response to different CO_2_ concentrations. And, the genetic reasons for the higher growth rate, CO_2_ fixation rate, and photosynthetic efficiency of domesticated microalgae cells under higher CO_2_ concentration remain unclear.

Nevertheless, CCM models have clearly shown that CCM will work when microalgae cells are exposed to limited CO_2_ conditions [14]. There is still a lack of research on the exact conditions, including CO_2_ conditions and *K*_m_ (CO_2_), and under which condition CCM would switch on. Moreover, the relationship between the diversity and evolutionary pathways of key carbon fixation-related genes and photosynthetic performance is still unknown because of their dependency on microalgae species [15]. Therefore, in the present study, we analyzed the transcriptome and gene expression of *Chlorella* PY-ZU1 cells cultivated under different CO_2_ concentrations and reconstructed the CO_2_ transport pathways into *Chlorella* PY-ZU1 responses to different CO_2_ concentrations. The growth conditions of *Chlorella* PY-ZU1 and DIC in the medium were also measured to extrapolate the value of *K*_m_ (CO_2_).

## Methods

### Strains and media

This strain used in the present study was *Chlorella* PY-ZU1, a highly CO_2_-tolerant and fast-growing microalgal species that obtained from *Chlorella pyrenoidosa* after γ irradiation and high CO_2_ domestication [6]. The cells were maintained and cultivated in Brostol’s solution (also known as soil extract, SE) [1, 6].

### Analysis of differentially expressed *Chlorella* PY-ZU1 genes under continuous aeration with 15% CO_2_ and air

*Chlorella* PY-ZU1 strains were cultivated in SE medium under 15% CO_2_ or air. Cells in the logarithmic phase (after cultivated 36 h) were collected by centrifugation for DNA extraction. The gene for full-length 18s rDNA was amplified to obtain the algal genome according to the protocol performed in Cheng’s study [16]. The following primers were utilized to amplify 18s rDNA: 18s-F, AACCTGGTTGATCCTGCCAGT and 18s-R, TGATCCTTCTGCAGGTTCACCT. The gene was inserted into the cloning vector, pMD19-T. Positive results were selected for sequencing. Total RNA was extracted by TRIzol reagent (Invitrogen) for cDNA library construction and Illumina sequencing. mRNA was separated by magnetic sand method, cleaved to synthesize double-stranded cDNA, and filled to plane. Poly (A) was added at the 3ʹ terminal end, and index connection was linked using TruSeq™ RNA Sample Preparation Kit. The target strip was enriched using polymerase chain reaction (PCR; 15 cycles) and recycled by 2% agarose gel. Quantitative determination was performed by TBS380 (Picogreen). Bridge amplification was conducted to generate cBot clusters. The 2*100 bp sequencing test was performed by HiSeq 2000 sequencing platform. Sequence assembly and annotation were similar to those performed in Cheng’s study [16].

### Gene expression statistics and differential expression analysis

Total RNA extracted from algae grown in normal medium (under Air) and high-CO_2_ medium [15% (v/v) CO_2_] was used to prepare gene expression libraries using the Illumina Gene Expression Sample Prep Kit and then subjected to Illumina sequencing. The RNA-Seq reads were mapped to our transcriptome reference database, and transcript abundances were quantified by RSEM (http://deweylab.biostat.wisc.edu/rsem/). Genes with differential expression between these two samples were identified using the numbers of mapped reads as EdgeR inputs (http://www.bioconductor.org/packages/release/bioc/html/edgeR.html). Genes were defined as differentially expressed if they exhibited a 2-fold or greater change between the air and high-CO_2_ samples and a false discovery rate (FDR) of 5% or less. Differentially expressed genes were regarded as up-regulated if their expression levels in high-CO_2_ samples were significantly higher than those in air samples. Conversely, genes that showed lower expression levels in the high-CO_2_ samples were regarded as down-regulated. Gene set enrichment analyses were performed using goatools (https://github.com/tanghaibao/goatools) and KOBAS (http://kobas.cbi.pku.edu.cn/home.do).

### Quantitative real-time PCR (qRT-PCR) validation

To investigate the developmental expression patterns of rubisco, CAs, and nitrate reductase, samples of microalgae cells cultivated for different durations under 15% CO_2_ or air were collected. Real-time reverse transcript polymerase chain reaction (real-time PCR, RT-PCR) was conducted with gene-specific primers pairs designed by PRIMER PREMIER5 software. The sequences of the specific primer sets are listed in Table 1. Total RNAwas extracted. qRT-PCR was performed with 20-μL reaction volumes containing 2 μL of 10-fold diluted cDNAs, 1 μM of each primer, and 10 μL SYBR Green Premix Ex Taq by the Bio-Rad Real-time PCR system (Bio-Rad, Hercules, CA, USA). The housekeeping gene for 18S ribosome DNA was used as a control. The 18 rDNA gene primers were as follows: algae sense 5′-ACGGCTACCACATCCAAG-3′ and antisense 5′-CCACCCGAAATCCAACTA-3′. The optimized qPCR program consisted of an initial denaturation step at 95 °C for 30 s, followed by 40 cycles at 95 °C for 5 s, and 60 °C for 30 s. qPCR was repeated thrice per gene. Each replication was performed with an independently prepared RNA sample and consisted of three technical replicates . A relative quantitative method (ΔΔCt) was used to evaluate the quantitative variation [17].

**Table 1:** Sequences of specific primers used for real-time PCR

Gene	EC no.	Sense	Antisense
Rubisco	4.1.1.39	CTCCACCCGCTCCGTCTAAG	GACAAACTCGTGCGACATTCTT
Nitrate reductase	1.7.1.1	GGGATGGGCGACCTTGAT	GCCTCCCGAACCTTGAGAA
CA	4.2.1.1	TGTGAGCGGACAGCAACCA	GGGACGAAGAGGAGAAGAGGG

### Cultivation of microalgae under continuous aeration with different concentrations of CO_2_

All *Chlorella* PY-ZU1 cultivation experiments were performed in an artificial greenhouse at 27 °C. Initial biomass concentration was maintained at 0.2 g L^−1^. Microalgae were cultivated in the cylindrical photoreactors (BR) (160 × Ф56 mm; 300-mL working volume) with the optimized SE medium (SE* medium). SE* medium contained 1 g of NaNO_3_, 0.15 g of K_2_HPO_4_·3H_2_O, 0.15 g of MgSO_4_·7H_2_O, 0.025 g of CaCl_2_·2H_2_O, 0.025 g of NaCl, 40 mL of soil extract, 0.005 g of FeCl_3_·6H_2_O, 1 mL of Fe-EDTA, and 1 mL of A5 solution in 958 mL of deionized water [1]. Enriched CO_2_ gas [from 384 ppm to 60% (v/v)] was bubbled into the BR via a pipe at a rate of 30 mL min^−1^. Initial pH was adjusted to 6.5 by using 0.1 M HCl and 0.1 M NaOH. During incubation, light intensity of 6000 Lux was applied on the surface of the BR with four cool white lights and two plant lights (TLD 36 W; Philips) fixed above the BR.

To obtain the dry biomass during cultivation, 10-mL samples were dewatered by centrifugation (Beckman Avanti J26-XP, USA) at 8500 rpm for 10 min and dried at 70 °C for 24 h. Biomass yield (g L^−1^) was calculated from the microalgae dry weight produced per liter. Chlorophyll was extracted by macerating microalgae in DMSO/80% acetone (1/2, V/V) and then measured [18]. NO_3_^−^ concentrations were analyzed by ion chromatography (MagIC, Metrohm, Switzerland). All experiments were performed in duplicate, and all data showed were reported as mean values and standard deviations (in figures) or standard errors (in tables) in this study.

### Calculation of dissolved CO_2_ concentration

Dissolved inorganic carbon (DIC) and CO_2_ in the culture were calculated according to the CO_2_ dissolved process [17] as follows:

$${\text{CO}}_{2} \,({\text{g}}) + {\text{H}}_{2} {\text{O}} \leftrightarrow {\text{H}}_{2} {\text{CO}}_{3} \,({\text{g}})\quad K_{\text{H}} = \frac{{\left[ {{\text{H}}_{2} {\text{CO}}_{3} } \right]}}{{{\text{P}}_{{{\text{CO}}_{2} }} }}$$

$${\text{H}}_{2} {\text{CO}}_{3} ({\text{g}}) \leftrightarrow {\text{H}}^{ + } + {\text{HCO}}_{3}^{ - } ({\text{g}})\quad K_{\text{a1}} = \frac{{[{\text{H}}^{ + } ][{\text{HCO}}_{3}^{ - } ]}}{{\left[ {{\text{H}}_{2} {\text{CO}}_{3} } \right]}}.$$

During cultivation, the culture pH was 5.5–7.0, thus,

$$[{\text{DIC}}] = [{\text{H}}_{2} {\text{CO}}_{3} ] + [{\text{HCO}}_{3}^{ - } ] = K_{\text{H}} \times P_{{{\text{CO}}_{2} }} \times \left( {1 + \frac{{K_{\text{a1}} }}{{{\text{H}}^{ + } }}} \right)$$

1 $$[{\text{CO}}_{2} ] = K_{\text{H}} \times P_{{{\text{CO}}_{2} }} .$$

The influent and effluent CO_2_ concentrations were monitored online by a CO_2_ analyzer (Servomex4100, UK). At a constant temperature of 27 °C, *K*_H_ = 3.2 × 10^−2^ M atm^−1^ and *K*_a1_ = 4.3 × 10^−7^ M [19]. [H^+^] = 10^−pH^, $P_{{{\text{CO}}_{ 2} }} = {\text{mean }}\left( {P_{{{\text{CO}}_{ 2} {\text{input}}}} , \, P_{{{\text{CO}}_{ 2} {\text{output}}}} } \right).$

### Reconstruction of inorganic carbon transport pathways

The microstructure of *Chlorella* PY-ZU1 was also measured by TEM in our previous study [20]. An apparent protein body (pyrenoid) was found inside the chloroplast in *Chlorella* PY-ZU1 from the image. The microstructure of *Chlorella* PY-ZU1 used in this work were compared with that of the green microalgae *Chlorophyta,* one species with the same genus of *Chlorella* PY-ZU1. In the Chlorophyta a pyrenoid is localized inside the chloroplast, starch often is accumulated around the pyrenoid and the presence of concentric thylakoid systems is visible around this starch sheath [21]. CCM goes on the pyrenoid model, and CA is in the pyrenoid that localized in chloroplast [22]. CCM goes on passive diffusion of CO_2_ through the plasmalemma and active bicarbonate transport into the chloroplast [8, 21]. Inorganic carbon transport pathways reconstructed based on de novo assembly and annotation of *Chlorella* PY-ZU1. The genes involved in these pathways in *Chlorella* PY-ZU1 were condensed and simplified according to their transcript abundances.

## Results

### Gene expression of carbonic anhydrase and rubisco under high CO_2_ stress

*Chlorella* PY-ZU1 had a higher biomass yield (2.85 g L^−1^) and shorter growth cycle (7 days) when cultivated in SE medium under continuous aeration with 15% (v/v) CO_2_ gas. This CO_2_ concentration is equivalent to that of flue gas from most coal-fired power plants. By contrast, the biomass concentration of *Chlorella* PY-ZU1 was only 1.30 g L^−1^ after 10 days of cultivation under air. It was previously reported that high CO_2_ induced algae growth [23]. Carbon fixation and nitrogen metabolism are the two most important aspects of primary cell metabolism. Therefore, we expected to observe significant differences in the expression of genes encoding enzymes of carbon fixation and nitrogen metabolism.

**Fig. 1:**
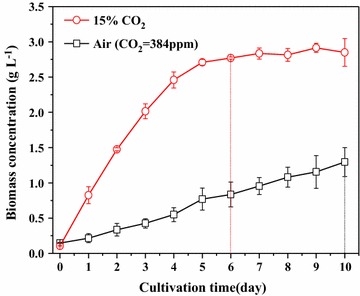
Growth curves of *Chlorella PY*-*ZU1* cultivated under continuous aeration with 15% CO_2_ gas versus air (CO_2_ = 384 ppm)

**Fig. 2:**
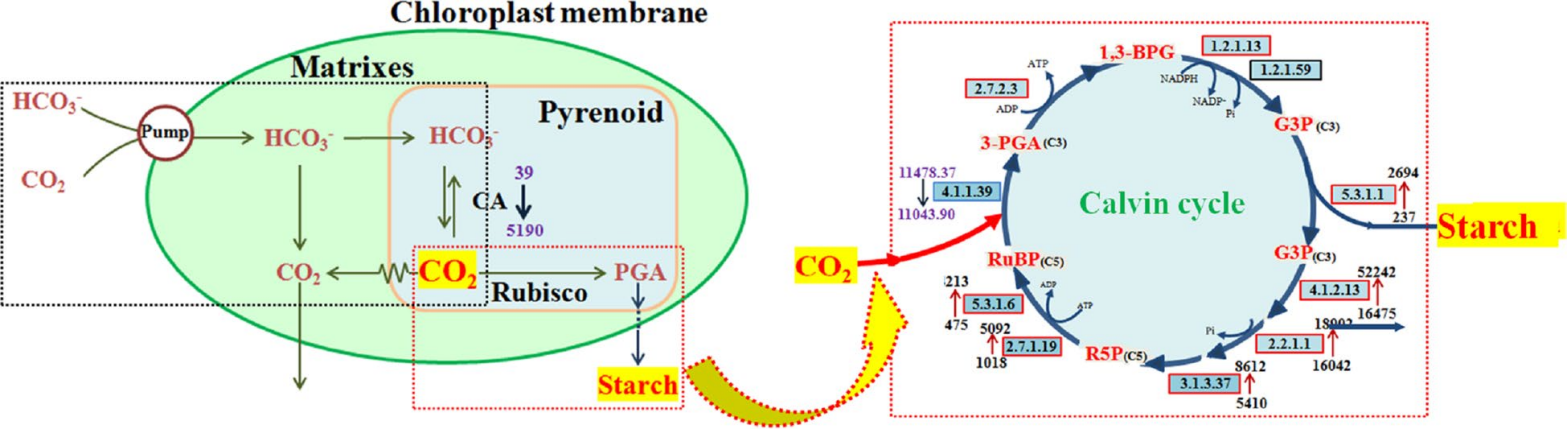
Genes transcript abundance of reconstructed carbon fixation pathway in *Chlorella PY-ZU1* cells cultivated with 15% CO_2_ gas versus air. *Black dotted box* indicated CO_2_ transfer process (CO_2_ concentrating mechanism), and *red dotted box* indicated Calvin cycle. Key enzymes of Calvin cycle are shown in *boxes* as enzyme commission (EC) numbers. *Red box* indicated an up-regulation, and *black box* indicated no significant changes

When aerated into the microalgal suspension, CO_2_ first dissolves in the medium, and then transfers from the extracellular culture medium through the cell membrane to the intracellular chloroplast. Then, CO_2_ is converted by ribulose-1,5-bisphosphate (RuBP) upon the catalysis of RuBP carboxylase (rubisco) to 3-phosphoglycerate (PGA), the precursor of structural materials in microalgal cells [24]. Rubisco (rbcS, EC4.1.1.39), the first enzyme of the Calvin cycle, fixes CO_2_ into the three carbon atoms of RuBP (${\rm CO}_{2} + {\rm C}5\mathop{\longrightarrow}\limits^{{\rm Rubisco}}2{\rm C}3$). The transcript abundance of RuBP increased slightly from 11,043.90 to 11,478.37 under high CO_2_ (15% CO_2_), whereas it was highly expressed under both high (15% CO_2_) and low (air) CO_2_ (Fig. 2). A 2-fold increase in transcript abundance was observed for PGK (EC2.7.2.3), which catalyzes the phosphorylation of 3-PGA to 1,3-bisphosphoglycerate. Moreover, triose phosphate isomerase (tpiA, EC5.3.1.1), which reversibly converts GAP into dihydroxyacetone phosphate (DHAP), increased by approximately 10.4-fold. The transcript abundance of a series of enzymes that converts GAP to sedoheptulose-7-phosphate (S7P), such as fructose-1,6-bisphosphatase aldolase (fbaB, EC4.1.2.13), increased more than 2-fold, whereas those of transketolase (tkt, EC2.2.1.1) and sedoheptulose-1,7-bisphosphatase (SBPase, EC3.1.3.37) improved slightly. Ribose-5-phosphate isomerase (rpiA, EC5.3.1.6), which converts R5P into ribulose-5-phosphate (Ru5P), increased by 7.8-fold. Phosphoribulokinase (prkB, EC2.7.1.19), which phosphorylates Ru5P into RuBP, increased by 4.0-fold. Therefore, almost all of the enzymes involved in the Calvin cycle had increased transcript abundances (Additional file 1) under high CO_2_. The results indicated that the whole carbon fixation process was driven by 15% CO_2_ gas. Therefore, the growth rate of *Chlorella* PY-ZU1 increased under 15% CO_2_ compared with under air (Fig. 1).

Moreover, rubisco was still highly expressed even *Chlorella* PY-ZU1 was cultivated under limited CO_2_, such as air. The high expression of rubisco was caused by CCM in microalgae cells. Rubisco is only activated when CO_2_ concentration is greater than its *K*_m_ (CO_2_), because that CO_2_ is the only carbon source that rubisco can utilize. However, when aerated with air, 99% of carbon in the culture is in the form of HCO_3_^−^ [1]. CO_2_ concentration in the medium hardly meets the requirements of rubisco because of the low solubility of CO_2_. Gene transcript abundance of CAs in *Chlorella* PY-ZU1 pyrenoids increased to 5190 from 39 under cultivation with 15% CO_2_. CAs expression increased to maintain high CO_2_ concentration in pyrenoids. Furthermore, CCM was simultaneously activated as most of the dissolved inorganic carbon, HCO_3_^−^, was transferred by pump through the chloroplast membrane into the internal pyrenoid; HCO_3_ was then converted by CAs to CO_2_ as function (2) in the chloroplast to meet the needs of rubisco (Fig. 2) [25]. However, CCM occurred at the cost of ATP. Increased CCM expression consumed more energy for CO_2_ transfer, thus decreasing the energy available for carbon fixation and other pathways of photosynthetic growth. Conversely, when cultivated under continuous aeration with 15% CO_2_, CAs was barely expressed in *Chlorella* PY-ZU1 pyrenoids. CCM was inactive. The CO_2_ that diffused directly into pyrenoids by high CO_2_ osmotic pressure was sufficient for rubisco. Therefore CO_2_ transfer pathway was simplified. Hence, more ATP was available for photosynthesis to promote growth.

2 $${\rm HCO}_{3}^{ - } + {\rm H}^{ + } \mathop{\longrightarrow}\limits^{{{\text{Carbonic anhydrase}}}}{\rm CO}_{2} +{\rm H}_{2} {\rm O}$$

**Fig. 3:**
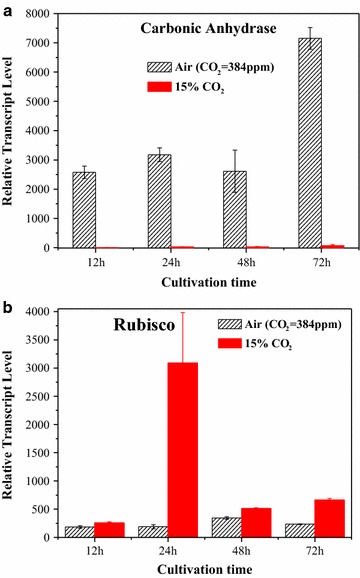
qRT-PCR of carbonic anhydrase (**a**) and rubisco (**b**) in *Chlorella PY*-*ZU1* cells that cultivated with 15% CO_2_ gas versus air

CAs and rubisco are the most important genes of carbon fixation pathways. To confirm the transcriptome results, the expression levels of genes encoding CA and rubisco were measured by qRT-PCR under different cultivation times (Fig. 3a). Under high CO_2_, the CAs transcript level was significantly reduced to 0 (The original data showed in Additional file 2), which is consistent with the transcriptome results (Fig. 2). Moreover, the study conducted by Fan et al. reported a similar, remarkably decreased CAs expression when oleaginous *Chlorella* cells were exposed to 5% CO_2_ [26]. This result is also consistent with previous reports that CO_2_ concentration significantly affects CAs activity in *Chlorella pyrenoidosa* cells, and that elevating CO_2_ concentration decreases CAs activity [27].

Rubisco expression levels increased under high CO_2_ (Fig. 3b). This result is consistent with the transcriptome results. Under 15% CO_2_, the enhanced rubisco expression of *Chlorella* PY-ZU1 cells might induce more CO_2_ to directly permeate intracellular pyrenoids for conversion into cellular energy storage molecules and to promote ATP conversion to glucose, thereby improving the photosynthetic efficiency of microalgae. Therefore, high CO_2_ concentrations reduced the ATP consumption of CO_2_ transfer. On the other hand, high CO_2_ concentrations improved CO_2_ conversion and photosynthetic efficiency, thus eventually reducing the growth cycle and increasing the biomass yield (2.85 g L^−1^) of *Chlorella* PY-ZU1.

In addition, the time when rubisco had highest transcriptional level (24 h of high CO_2_ and 48 h of air) and the time CAs had lowest transcriptional level under air (48 h) were also the time when maximum microalgae growth rate was achieved (24 h of high CO_2_ and 48 h of air) (Fig. 1). That fully illustrated that CAs and rubisco had the ability to regulate microalgae growth.

### Response of nitrogen metabolism and chlorophyll synthesis to high CO_2_ stress

**Fig. 4:**
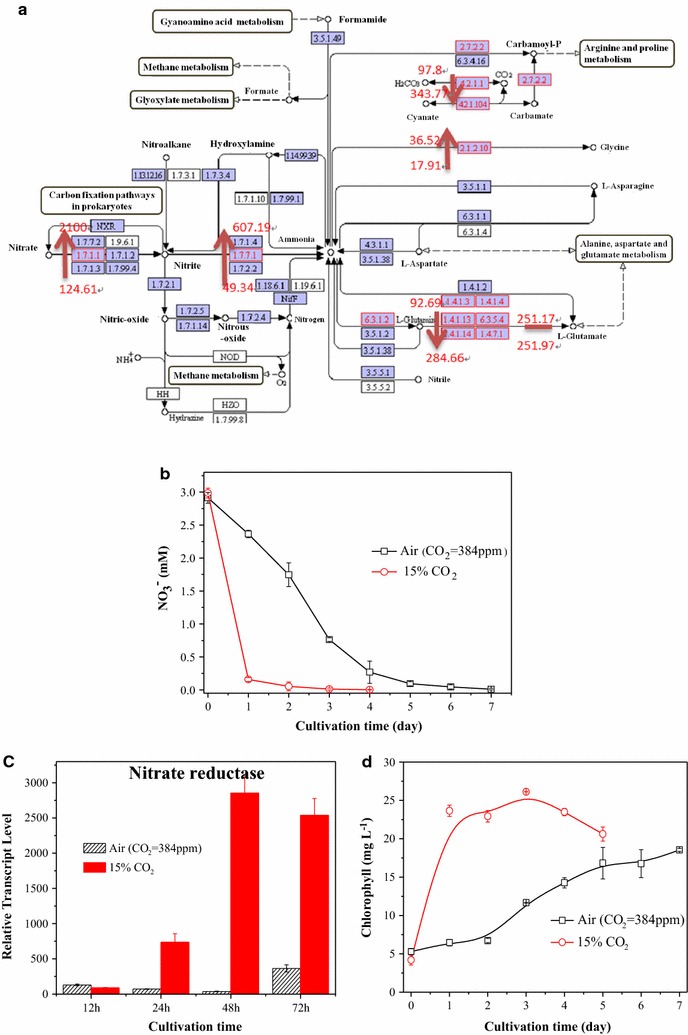
Genes transcript abundance of nitrogen metabolism pathway in cells (**a**), nitrate consumption (**b**), qRT-PCR of nitrate reductase (**c**) and chlorophyll synthesis (**d**) of *Chlorella* PY-ZU1 cultivated under 15% CO_2_ versus air. Key enzymes of Calvin cycle were shown in *boxes* as enzyme commission (EC) numbers. *Red box* indicated an up-regulation, *blue box* indicated a down-regulation, and *black box* indicated no significant changes

Similar to carbon fixation, the transcript abundance of important enzymes in nitrogen metabolism, including those of nitrate reductase, nitrite reductase and glutamate dehydrogenase (ghdA), also increased under high CO_2_ (Fig. 4a). Nitrogen is an essential element for chlorophyll and protein synthesis. After nitrate reductase and nitrite reductase increased, nitrate ions absorbed by microalgae were immediately catalyzed to nitrite ions and then to ammonia through a series of reduction reactions to synthesize nitrogenous compounds, such as amino acids, which in turn increased the efficiency of nitrate ion uptake from the medium by microalgae cells. On the aspect of nitrogen source consumption (in this study, the nitrogen source was sodium nitrate), *Chlorella* PY-ZU1 consumed almost all of the 3 mM nitrate during the first 2 days, especially on the first day (Fig. 4b) when cultivated under continuous aeration with 15% CO_2_. By contrast, *Chlorella* PY-ZU1 only consumed 1.26 mM nitrate during the first 2 days, and nitrate was not completely consumed until the sixth day when cultivated under air. On the genetic level, the transcript abundance of nitrate reductase, the first enzyme in nitrogen metabolism, increased by approximately 10-fold by the 24th h and 8-fold by the 48th h under high CO_2_ compared with that under air (Fig. 4c). The higher transcript expression of nitrate reductase accelerated the transformation of nitrate to nitrite and O_2_ catalyzed by nitrate reductase (Function 3). This higher gene expression might manifest by the rapid consumption of nitrate. Furthermore, by the catalysis of the up-regulated nitrite reductase, more nitrite was converted to amino acid synthesis precursors, such as ammonia (Function 4), thereby accelerating the synthesis of proteinaceous materials, such as chlorophyll (Fig. 4d).

3 $$2{\rm NO}_{3}^{ - } \mathop{\longrightarrow}\limits^{{\rm Nitrate}\,\, {\rm reductase}}2{\rm NO}_{2}^{ - } + {\rm O}_{2}$$

4 $${\rm NO}_{2}^{ - } + 6{\rm NADPH}\mathop{\longrightarrow}\limits^{{\rm Nitrite}\,\,{\rm reductase}}{\rm NH}_{4}^{ + } + 2{\rm OH}^{ - } + 6{\rm NADP}$$

Under 15% (v/v) CO_2_, 23.65 mg L^−1^ of chlorophyll was produced during the first day of cultivation. Chlorophyll concentration remained stable in the range of 23–26 mg L^−1^. All of the 3 mM nitrate in the medium was consumed during the following 3 days (Fig. 4d). Chlorophyll was vital in photosynthesis and allowed *Chlorella* PY-ZU1 cells to absorb energy from light. Moreover, light conversion efficiency is linearly correlated with chlorophyll content [1, 28]. Increased chlorophyll provided more energy for photosynthetic reactions, thereby improving the photosynthetic growth rate of *Chlorella* PY-ZU1. However, the high nitrogen consumption during the first 3 days resulted in nitrogen deficiency in the following days under 15% CO_2_. Microalgae consumed its chlorophyll to maintain cell growth under nitrogen deficiency from the 4th day, and the chlorophyll content of *Chlorella* PY-ZU1 decreased. By contrast, chlorophyll content of *Chlorella* PY-ZU1 still increased when cultivated under air. Given that chlorophyll synthesis is almost directly proportional to nitrate concentration in the culture medium, the chlorophyll contents of *Chlorella* PY-ZU1 were almost the same under different CO_2_ conditions by the end of the cultivation period [1, 29]. However, during cultivation, the chlorophyll content of *Chlorella* PY-ZU1 cultivated under 15% CO_2_ was always higher than that of under air, which resulted in higher microalgae growth rate (Fig. 1).

### Analysis of different concentrations of CO_2_ transport and fixation mechanisms

**Fig. 5:**
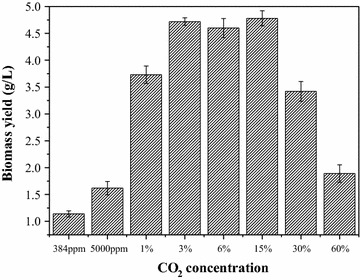
Biomass yield of *Chlorella* PY-ZU1 cultivated with optimized SE medium under various concentrations of CO_2_

Figure 5 shows the biomass productivity of *Chlorella* PY-ZU1 cultivated in optimized SE medium under different CO_2_ concentrations. Excessively low (<1%) and high (>30%) CO_2_ concentration could restrain microalgae growth and resulted in lower biomass yield (<2 g L^−1^). However, when cultivated under 1% CO_2_, the biomass yield drastically increased by 130.2% to 3.73 g L^−1^ compared with the 1.62 g L^−1^ obtained by cultivation under 0.5% CO_2_. The drastically increased biomass yield in response to higher CO_2_ concentrations indicated some changes in the pathway of CO_2_ transfer and utilization by microalgae. CCM will work when microalgae is cultivated under limited CO_2_ conditions, such as air. However, it will not work if the CO_2_ that directly diffused to pyrenoids by extra- and intracellular CO_2_ osmotic pressure is sufficient for rubisco, more energy was concentrated for cell growth. Thus, biomass productivity was dramatically enhanced [11]. An external concentration of 0.5% CO_2_ was too low for *Chlorella* PY-ZU1 because it could not supply enough CO_2_ through osmosis to rubisco. However, 1% external CO_2_ could overcome diffusion resistance to enable CO_2_ flux from the external medium to the cytoplasm. Moreover, enough CO_2_ diffusion by high CO_2_ stress to the cytoplasm resulted in non-operational CCM. Therefore, >1% external CO_2_ concentration maintained enough CO_2_ in pyrenoids for rubisco only through direct diffusion, which is dependent on extra- and intra-cellular CO_2_ osmotic pressure. When cultivated under 3–30% CO_2_, *Chlorella* PY-ZU1 had stable, higher biomass yields of 4.60–4.78 g L^−1^. Therefore, 3–15% was ideal CO_2_ concentration for *Chlorella* PY-ZU1 growth. However, when CO_2_ concentration exceeded 30%, excess CO_2_ inhibited microalgal growth and sharply decreased biomass yield (1.89 g L^−1^). Therefore, >30% external CO_2_ is too high for *Chlorella* PY-ZU1 given the overly acidic culture after aeration with higher CO_2_ concentrations [26, 27].

## Discussion

The CCM process has a specific CAs to catalyze the conversion of HCO_3_ to CO_2_ at the expense of ATP [26]. For optimal efficiency, the carboxysomal CAs activity needs to match, as closely as possible, the maximal rate of CO_2_ fixation [4, 30] under limited CO_2_ conditions. The dissolved CO_2_ concentration increased from 6.14 μM to 4.60 mM as the aerated CO_2_ concentration increased from 384 ppm (air) to 15% (Table 2). *K*_m_ (CO_2_) of rubisco is the critical point in the activation of rubisco [15] and allows cells to initiate the CCM process [31]. Therefore, microalgal growth rate and biomass yield will increase significantly when the dissolved CO_2_ concentration in the culture is higher than *K*_m_ (CO_2_) of rubisco. The biomass yield of *Chlorella* PY-ZU1 cultivated under 1% CO_2_ increased sharply by 130.2% to 3.73 g L^−1^ compared with 1.62 g L^−1^ obtained from cultivation with 0.5% CO_2_. It is known that CCM is energy consumption process and *K*_m_ is the critical point in the activation of rubisco. On one hand, if the intracellular CO_2_ concentration around rubisco is higher than *K*_m_, CCM would not work. More energy would be saved for microalgae growth. This is to say we can estimate whether CCM works from the microalgae growth condition. On the other hand, the mixed CO_2_ gas aerated into microalgae suspension as carbon source. The intracellular CO_2_ should be balanced with the dissolved CO_2_ in suspension without CCM after equilibrium of CO_2_ gas dissolving. The microalgae growth rate has a significant increase when the dissolved CO_2_ concentration in culture increased from 80 to 192 μM. It could be deduced CCM did not work under 192 μM of dissolved CO_2_ concentration but might still work under 80 μM. Therefore, the *K*_m_ (CO_2_) value of rubisco in *Chlorella* PY-ZU1 might be in the range of 80–192 μM, which corresponds to 1% aerated CO_2_ concentration. These values are consistent with those of previous reports.

**Table 2:** pH and dissolved CO_2_ concentration of *Chlorella* PY-ZU1 culture aerated with various concentrations of CO_2_ gas

Aerated CO_2_ conc. %	0.0384	0.5	1	3	6	15	30	60
pH	10.73 ± 0.15	9.86 ± 0.41	8.75 ± 0.10	7.88 ± 0.07	7.35 ± 0.05	6.96 ± 0.17	6.04 ± 0.58	5.63 ± 0.38
Dissolved CO_2_ conc. (μM)	6.14 ± 0.04	80 ± 2.0	192 ± 1.8	800 ± 20	1600 ± 32	4600 ± 20	9400 ± 37	19,100 ± 56

**Fig. 6:**
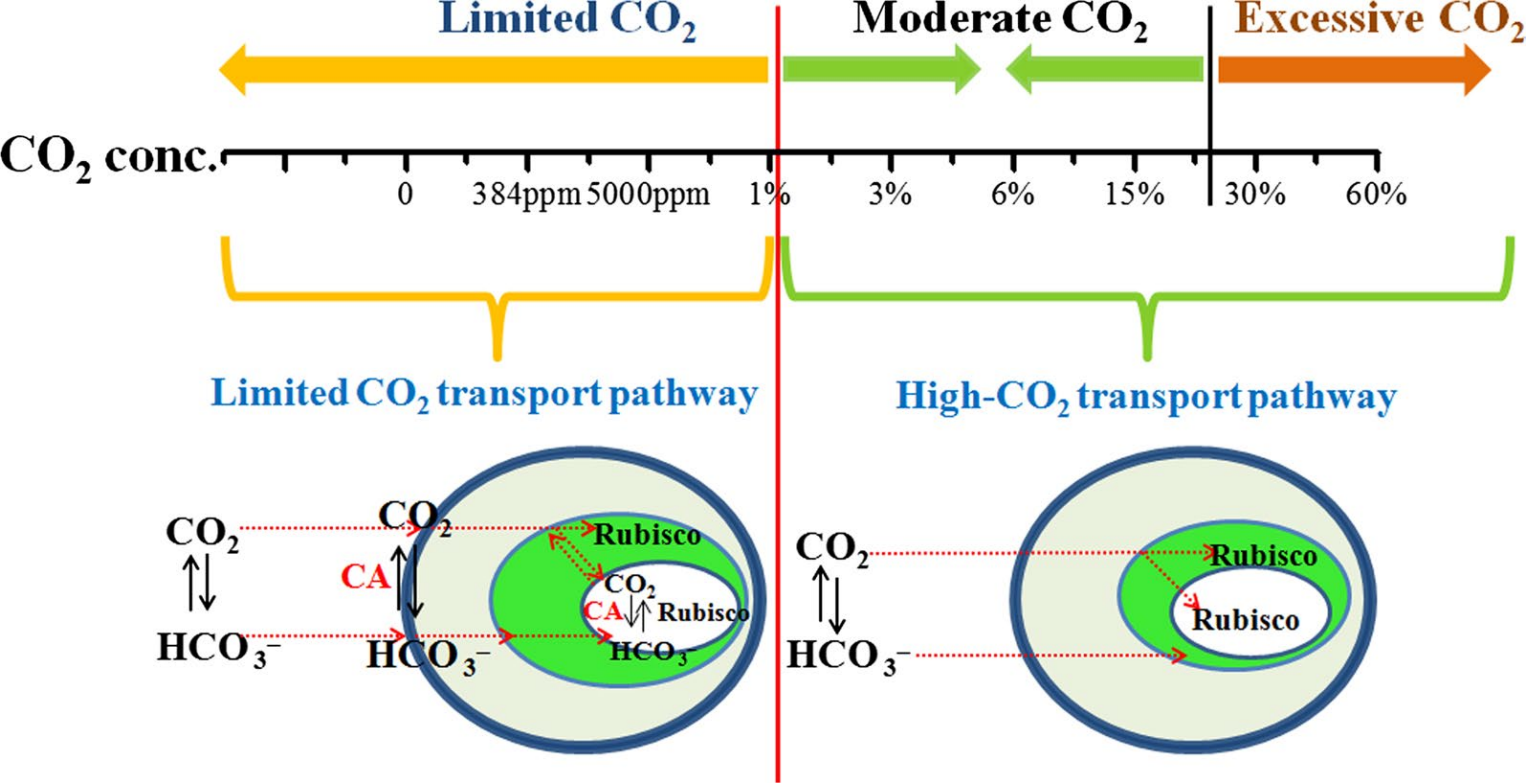
Reconstructed CO_2_ transport schematic pathway of *Chlorella* PY-ZU1 under various concentrations of CO_2_

Furthermore, we utilized de novo assembly and annotation to reconstruct the possible pathways in *Chlorella* PY-ZU1 cells for inorganic carbon transport to pyrenoids from the external medium in response to different aerated CO_2_ concentrations based on the pyrenoid model [22]. When cultivated under limited CO_2_, such as under air, the dissolved CO_2_ concentration of 6.14 μM in the culture could not meet the CO_2_ demands of rubisco. Therefore, *Chlorella* PY-ZU1 initiated CCM to localize CO_2_ in pyrenoids. However, >1% CO_2_ concentration supplied enough dissolved CO_2_ (>192 μM). Thus, the cell did not initiate CCM. The free CO_2_ molecules permeating to the pyrenoids were sufficient for direct use by rubisco. Increased CO_2_ accumulation increased the cell’s photosynthetic efficiency, resulting in a higher biomass yield of 3.73 g L^−1^. In addition, with the further increasing of CO_2_ concentration to 30%, the dissolved CO_2_ concentration increased to 9.4 mM. Although, the dissolved CO_2_ also could meet the demands of rubisco. Excessive CO_2_ could acidify the microalgae culture, resulting in cell “anesthesia” [32] and decreasing enzymatic activity [33, 34], which eventually drastically decreases *Chlorella* PY-ZU1 biomass yield. Therefore, <1% CO_2_ was too limited for *Chlorella* PY-ZU1. As a result, CCM was initiated to concentrate CO_2_. Therefore, 1–30% CO_2_ concentration is very suitable for *Chlorella* PY-ZU1 growth, whereas >30% CO_2_ is excessive for microalgae growth (Fig. 6).

## Conclusions

The biomass yield of *Chlorella* PY-ZU1 cultivated under 0.5% CO_2_ was only 1.62 g L^−1^. However, when cultivated under 1% CO_2_, the biomass yield drastically increased by 130.2% to 3.73 g L^−1^. The drastically increased biomass yield in response to higher CO_2_ concentration indicated some changes happened in CO_2_ transfer and utilization by microalgae. CAs in *Chlorella* PY-ZU1 cells cultivated under 15% CO_2_ barely expressed. That indicated CO_2_ could directly permeate into intra-cell for rubisco to fix without CCM working at the expense of ATP. While CCM in cells that cultivated under <1% CO_2_ conditions would work for CO_2_ transport. Carbon fixation and nitrogen metabolisms are the two most important aspects of primary cell metabolisms. Besides the improved expression of enzymes in carbon fixation pathways, the enzymes in nitrogen metabolism pathways, including nitrate reductase, nitrite reductase and glutamate dehydrogenase, also had an increased expression level under 15% CO_2_ condition. Further studies should be taken to determine CO_2_ distribution and verify the exact *K*_m_ (CO_2_) of rubisco in microalgae cells.

## Additional files

**Additional file 1.** Genes differental expression of *Chlorella* PY-ZU1 under 15% CO_2_ compared with that under air.

**Additional file 2.** Original data of qRT-PCR.

## Abbreviations

CAs: carbonic anhydrase
CCM: CO_2_ concentrating mechanism
*K*_m_ (CO_2_): concentration of CO_2_ required for 50% of the maximum photosynthesis rate
Rubisco: ribulose-1,5-bisphosphate carboxylase/oxygenase
RuBP: ribulose-1,5-bisphosphate
3-PGA: 3-phosphoglyceride
DHAP: dihydroxyacetone phosphate
S7P: sedoheptulose-7-phosphate
fbaB: fructose-1,6-bisphosphatase aldolase
Ru5P: ribulose-5-phosphate
SBPase: sedoheptulose-1,7-bisphosphatase
